# The econometric analysis of voluntary environmental regulations and total factor productivity in agribusiness under digitization

**DOI:** 10.1371/journal.pone.0291637

**Published:** 2023-09-14

**Authors:** Min Chen, Lili Zhang

**Affiliations:** School of Business, Wenzhou University, Wenzhou, Zhejiang, China; University of Cagliari: Universita degli Studi Di Cagliari, ITALY

## Abstract

Under the new development pattern, both "digital" and "low-carbon" development have entered the fast track, and digital transformation has become an important path to promote green development and enhance total factor productivity in agriculture. Based on the data of agricultural companies, this paper empirically verifies the impact of voluntary environmental regulations on total factor productivity. The empirical results show that voluntary environmental regulation has a significant positive impact on total factor productivity of agribusiness. In the mechanistic analysis, it is found that voluntary environmental regulations accelerate the digital transformation process of firms, which in turn increases their total factor productivity. In addition, the level of government environmental concern contributes to the increase of voluntary environmental regulations on firms’ total factor productivity. The findings have practical implications for the sustainable development of agribusiness, providing empirical evidence for policy formulation and adjustment, and helping the agricultural economy to achieve high-quality development.

## 1. Introduction

In the context of the current transformation of the economy from high-speed development to high-quality development, focusing on improving total factor productivity is an important task to promote high-quality economic development. At the same time, the improvement of total factor productivity is a key factor in China’s agricultural growth [1]. With the implementation and advancement of the rural revitalization strategy, the agricultural development model should be transformed from increasing factor inputs to improving total factor productivity. In the current strategic context, increasing total factor productivity in agriculture is a prerequisite for improving the living standards and a basis for ensuring national independence. Thus, improving the productivity and competitiveness of the country’s agriculture, and accelerating its transformation into a major agricultural country have become important goals. An increase in the total factor productivity of leading agricultural enterprises is the key to achieving a steady increase in agricultural productivity.

The development of the agricultural economy depends on the agricultural environment, and a good agricultural environment is a key element for agricultural production and sustainable development [2]. However, due to the long-term misuse of agricultural fertilizers and pesticides, excessive waste emissions from agricultural enterprises and urbanization, environmental problems such as soil erosion, declining fertility and agricultural surface pollution in the agricultural environment have become increasingly prominent [3]. In the 2020 Annual Report on China’s Ecological Environment Statistics, it is shown that the national chemical oxygen demand emissions from wastewater were 25.648 million tons, of which the chemical oxygen demand emissions from agricultural sources were as high as 15.932 million tons, and the national ammonia nitrogen emissions from wastewater were 984,000 tons, of which the ammonia nitrogen emissions from agricultural sources were 254,000 tons, which not only reflects the more serious pollution in China’s agriculture, but also reflects the fact that the intensity of environmental regulation in agriculture needs to be further increased. The prevention and control of agricultural surface pollution is an important tool for achieving high-quality agricultural development. To promote the control of agricultural surface pollution, it is crucial to effectively match the relevant government policies with environmental regulation tools.

Wang et al. [4] proposed that China’s economic construction has caused huge resource consumption and environmental damage. And appropriate environmental regulation helps to achieve the coordinated development of economic construction and ecological environmental protection. In recent years, with the promotion of ecological civilization and the implementation of the concept of green development, environmental-related policies and regulatory tools have become increasingly sophisticated [5]. The current types of environmental regulation tools can be divided into three categories: command-controlled environmental regulation, market incentive environmental regulation and Voluntary environmental regulation [6]. Voluntary environmental regulation projects are mostly initiated by governments, industry organizations and independent third-party organizations [7]. Voluntary participatory environmental regulation is more flexible and autonomous than command-controlled environmental regulation or market incentive environmental regulation. It not only helps to stimulate firms to innovate in order to promote the spontaneous provision of environmental public goods [8], but also effectively reduces the regulatory cost of the government, and alleviates the resistance role of the government in environmental governance to a certain extent.

Under the new situation of hard constraints on production factors and environmental carrying capacity, Cai and Ye [9] argue that as ecological constraints tighten, it has become a crucial issue to deal with the synergistic development of ecological environmental protection and economic growth. More and more scholars have conducted in-depth research on the relationship between environmental regulation and total factor productivity [10]. Yang et al. [11] argued that the relationship between environmental regulation and enterprise total factor productivity has been a hot topic in environmental economics research, but the research conclusions are still not uniform. After combing through the relevant literature, this paper finds that most of the literature analyses the research problem of environmental regulation and enterprise total factor productivity in the direction of industry, manufacturing industry or the whole industry. And there are fewer analyses directed at the agricultural industry. However, regulating environmental pollution in agricultural development and improving agricultural total factor productivity are important issues that need to be addressed in the current agricultural development. Therefore, unlike the focus of previous studies, this paper focuses on analyzing whether environmental regulation in agribusiness can help improve total factor productivity. It also analyses the mechanism from the perspective of digital transformation of enterprises, want to find out whether voluntary environmental regulation can drive digital transformation and thus have an impact on total factor productivity? Is there a win-win situation for environmental protection and productivity gains? These are the questions that need to be answered. The purpose of this study is to further expand the theory of the Porter’s Hypothesis by refining the research on environmental regulation and total factor productivity in the field of agriculture. On the practical side, it will provide policy recommendations for the sustainable development of agribusinesses and information for government policies related to the development of green agriculture.

The rest of the paper is organized as follows: Section Literature Review presents a literature review of the relationship between environmental regulation and total factor productivity of firms; Section Theoretical Analysis discusses the theoretical mechanisms and research hypotheses; Section Data and Variables presents the sample data and empirical model; Section Empirical Results Analysis presents the results of the empirical analysis of the association between voluntary environmental regulation and total factor productivity of agricultural firms; Section Conclusions and Further Discussion gives the conclusions and policy recommendations.

## 2. Literature review

In the early days, scholars believed that environmental regulation only included government intervention in environmental resources, In other words, the government set relevant environmental standards in order to restrict the production and management activities of enterprises. Subsequently, scholars have verified that economic incentives of market mechanism also play a regulating effect on environmental resources. Palmer et al. [12] argue that market-incentivised environmental regulation is superior to government-controlled environmental regulation, and Yumin Zhao et al. [6] extend their analysis to suggest that environmental regulation is essentially an individual or organisational approach to environmental protection, with the purpose of environmental protection and a tangible system or intangible awareness as the means. Subsequently, as companies became more aware of environmental governance, the emergence of environmental certification and voluntary agreements expanded the meaning of environmental regulation to include voluntary environmental regulation in addition to command-and-control and market-incentive environmental regulation [13]. Voluntary environmental regulation provides an independent approach and space for each company to set the most appropriate regulatory standards to improve its environmental performance according to its own characteristics and actual business situation [14]. This kind of incentive mechanism to encourage enterprises to carry out environmental protection behavior is helpful to fundamentally alleviate environmental pollution problems [15].

Total Factor Productivity, as a reflection of the core competitiveness of enterprises, is an important indicator for analyzing the dynamics of economic growth and evaluating the quality of economic growth. As regards the measurement of total factor productivity, Tibergen introduced the time factor into the Cobb-Douglas production function and introduced a more comprehensive concept of total factor productivity indicators for the first time. Subsequently, Solow’s pioneering work led to widespread interest in total factor productivity. According to the Solow theory of economic growth, total factor productivity is the increase in output resulting from factors such as technological progress, in addition to the contribution of the various production input factors. Research on total factor productivity has been the focus of scholars in related fields at home and abroad. From the macro level, scholars have carried out in-depth analysis on measurement methods [16, 17], regional differences [18], driving factors [19] and other aspects [20] of total factor productivity. Total factor productivity and the determinants of its growth are persistent research hot issues in academia [21], among which the factors that have received wide attention are financial development, international trade, R&D, infrastructure, human capital, etc. At the micro level, the existing literature focuses on the factors influencing the total factor productivity of firms, mainly based on data from industrial or manufacturing enterprises, combining R&D investment [22, 23], human capital [24], resource allocation [25], environmental regulation [26], technological innovation [27] and other factors [28, 29] for empirical analysis.

In exploring the relationship between environmental regulation and total factor productivity, scholars have carried out in-depth research on the effects of both using relevant theories and empirical analyses. The existing literature on the effect of environmental regulation on total factor productivity mainly focuses on the following three aspects. The first aspect is the negative effect of environmental regulation on total factor productivity. Labour productivity in the United States stagnated significantly in the 1970s, and Haveman [30] argues that environmental regulation was one of the factors that brought it to a standstill. Therefore, the traditional view is that environmental regulation leads to additional costs for firms, reducing their competitiveness in the marketplace and thus constraining their productivity [31]. Chen [32] also revalidates this view with data on Chinese industry, arguing that environmental regulation has a negative effect on the growth of industrial total factor productivity in China, and suggests that the increase in China’s total factor productivity is mainly due to technological progress. Further Li et al. [33] analysed from the perspective of urban green total factor productivity arguing that the implementation of environmental regulations significantly suppresses urban total factor productivity.

The second aspect is that environmental regulation has a positive impact on total factor productivity, and by the early 1990s, some scholars argued that environmental regulation has a positive spillover effect on productivity, resulting in a positive win-win effect. Porter [34] suggests that environmental regulation does not reduce the competitiveness of US industry in the international marketplace, but rather pushes firms to innovate. Accordingly, the "Porter’s hypothesis" theory emerged. Following this, Das et al. [35] based on US forestry data, Hamamoto [36], based on Japanese manufacturing data, and Qu and Xi [37] based on Chinese industrial data, found that environmental regulation has a significant effect on total factor productivity. In terms of mechanism analysis, innovation capacity plays an important indirect role between environmental regulation and productivity growth [38].Yan et al. [39] based on industrial data proposed that innovation capacity is an exogenous mechanism variable for environmental regulation to be used for green total factor productivity in industry. So that it suppresses industrial pollution emissions while increasing industrial productivity. Wang et al. [40] also found a significant mediating effect of regional innovation capacity between environmental regulation and green total factor productivity based on data from China’s logistics industry. From the direction of the three major types of environmental regulation, Peng et al. [41] showed that market-based environmental regulation has a significant productivity-enhancing effect on industrial firms. However, Feng et al. [42] through an econometric model concluded that market-based environmental regulation does not have a significant effect on total factor productivity while command-based environmental regulation can increase total factor productivity. Zhou et al. [43] suggested that there is a significant positive relationship between voluntary environmental regulation and total factor productivity in agriculture.

The third aspect is that environmental regulations have a non-linear effect on TFP, At first, Darnall et al. [44] verify that appropriate environmental regulations can improve firm performance, but stricter environmental regulations have a negative impact. Then, Sanchez-Vargas et al. [45], Liu et al. [46] in the study of firms’ total factor productivity recognized that the effect of environmental regulation on productivity is non-linear, and that appropriate intensity of environmental regulation can increase firms’ total factor productivity, but too much regulation can instead have an inverse effect on it. Stavropoulos et al. [47] argue that there is a ’U’ shaped relationship between environmental regulations and the competitiveness of China’s IC industry, with positive effects in the long run as long as the regulations are properly designed. Lu and Chen [48] based on data from the transport sector, found an intermediate value for the effect of environmental regulation intensity on total factor productivity growth. In addition, Tian and Feng [49] expanding on existing studies, examined the internal structure of different types of environmental regulation on green total factor productivity and showed that there is a significant non-linear relationship between different types of environmental regulation and the intrinsic factors of total factor productivity.

A review of the existing literature shows that scholars have further analyzed the impact of environmental regulation on enterprise productivity, competitiveness and socio-economic growth by presenting the possible effects of environmental regulation on the productive activities of enterprises from different perspectives. However, there is room for expansion in the research on environmental regulation and total factor productivity. Firstly, Most of the existing studies on the measurement of environmental regulation and total factor productivity are at the macro-provincial level and focus on industries, few scholars have focused on the impact of environmental regulation on the total factor productivity of agricultural enterprises. Secondly, most of the research on the relationship between environmental regulation and firms over the past two decades has remained based on the perspective of mandatory environmental regulation. However, in recent years, many scholars have noted the existence of voluntary environmental regulation by enterprises and have studied it. With the gradual implementation of the concept of agro-ecological civilization and green development, as well as the gradual increase in public awareness of environmental protection, voluntary environmental regulation policies in the form of environmental certification, environmental hearings and public participation have received increasing attention. Thirdly, most of the existing studies have focused on firms’ innovative capacity and resource allocation efficiency in their mechanistic analyses. But with the integration and innovation of digital technology in the energy and environmental sectors, the role of digitization in corporate environmental governance is also gaining attention. In the current context of synergistic development of ecological environment and economic growth, the question of whether voluntary environmental regulation helps to promote innovative activities and total factor productivity in agribusiness is a research question in line with the trend. This paper analyses the effect of voluntary environmental regulation on firms’ total factor productivity under the digital transformation path of agribusiness through an empirical model, and explores the role of the government’s level of concern for the environment as a player in it. In this way, it further extends the research on environmental regulation and firm development, and provides empirical evidence for dual environmental and economic growth from an agribusiness perspective.

## 3. Theoretical analysis

### 3.1 Corporate voluntary environmental regulation and total factor productivity

Agribusiness is a special industry that is both highly dependent on and directly influences the ecological environment. A good ecological environment is both a prerequisite for sustainable development and a source of competitive advantage for agribusinesses. In the relationship between environmental regulation and total factor productivity, under static resource constraints, environmental regulation increases firms’ costs, influences their input choices, reduces their innovation capacity [50], and distorts their resource allocation [51]. However, from a dynamic perspective, Porter (1991) suggests that the inverse relationship between environmental governance and productivity based on US industrial data is only derived from a static perspective and that environmental regulations do not reduce their international competitiveness [52]. Shen and Zhang [53] argue that environmental regulation has a significant effect on total factor productivity in various industries, and that environmental regulation and economic growth in China are now on a virtuous track of synergistic development. Based on Porter’s hypothesis, appropriate and reasonable environmental regulations can promote firms’ ability to improve their technological innovation, which can have an "innovation compensation" effect on firms [54], partially or even completely offset the negative effects of the cost burden of environmental regulations, improve environmental management and increase. This improves the competitiveness of firms and creates a ’win-win’ situation for environmental protection and total factor productivity.

At present, the types of environmental regulation can be divided into three categories: command-and-control, market-incentive and voluntary participation [6], and compared with command-and-control and market-incentive environmental regulation, the responsible subject of voluntary participation environmental regulation is the enterprise.

On the one hand, enterprises have more autonomy and choice in meeting environmental standards. On the other hand, according to the "compliance cost theory", voluntary environmental regulation imposes constraints on production and affects the costs of enterprises [55]. That is, if an enterprise completes environmental certification but is found to have falsified information or has been reported by the public for environmental problems, this will cause irreparable damage to the image of the enterprise and indirectly increase the cost of regulation for the enterprise. As a result. Hereby, firms’ awareness of environmental governance increases under the influence of environmental regulation, which helps them overcome organization inertia and optimism their production and business models [56]. According to the theory of planned behaviour, intentions influence the behavioral motivations of micro-subjects, and the stronger the behavioral intentions generated by a subject, the greater the likelihood of its implementation. If a company is certified under the ISO 14001 environmental management system, it is more likely to be willing to improve its production processes and strengthen its environmental management to achieve both environmental and economic benefits [57]. Therefore, the willingness of firms to voluntarily regulate the environment will drive them to actively seek innovation and productivity improvement paths. Based on the above analysis, this paper proposes the following hypotheses:

H1: Voluntary environmental regulation by agricultural firms has a catalytic effect on total factor productivity.

### 3.2 Digital transformation, voluntary environmental regulation and total factor productivity

In the current context of digital development, digital transformation is an important strategic tool for companies to achieve their sustainability goals [58]. Agricultural transformation helps companies to address issues and challenges and ensure sustainable agricultural development [59]. Depending on the impact of environmental regulation on a firm’s product differentiation, there are two main strategic options for firms. The first is the ’regulation-responsive’ approach, where companies simply follow simple environmental regulation requirements and take no other proactive measures. The second is the ’opportunity-seeking’ type, in which firms with a greater awareness of environmental governance improve their ability to differentiate their products through technological innovation in order to alleviate the pressure of increasing costs and thus improve their competitiveness [60]. In addition, voluntary environmental regulation will help firms to improve their own management and build a positive image of environmental concern, thereby increasing total factor productivity.

When the enterprise implements environmental regulation, its production costs will be directly affected, and Porter believes that appropriate environmental regulation can stimulate the enterprise’s "innovation compensation" effect, that is, the cost changes caused by environmental regulation will stimulate the enterprise’s technological innovation behaviour [61], which will increase the enterprise’s total factor productivity. Voluntary environmental regulation improves the total factor productivity of enterprises through digitization processes that enhance technological innovation, promote strategic transformation and restructure the organization of enterprises [62]. On the production side of the enterprise, after the enterprise has passed the environmental management system certification, it intervenes in the production of the enterprise as an internal constraint, which breaks the original production balance of the enterprise and changes the way of production inputs in order to meet the constraints. On the production side, in order to pass the certification and annual inspection, the voluntary environmental regulation will drive enterprises to widely apply digital information technology such as artificial intelligence, block-chain and cloud computing, create green production processes, and promote the research and development, innovation and application of key common technologies and leading-edge technologies in the fields of high energy efficiency, resource recycling, zero carbon energy and negative emissions, so as to improve productivity and production efficiency. On the management side, due to the changes in the production side of the enterprise, it has to adjust the factor inputs of the enterprise, thus changing the management style and management costs in the traditional operation of the enterprise, which will also further affect the productivity level of the enterprise. according to the continuous self-improvement of voluntary environmental regulations, the demand for enterprises to improve their environmental performance will prompt them to adjust their management models to meet the new production requirements, stimulate them to undergo digital transformation, build a management system supported by data, network sharing and intelligent collaboration, and further enhance the efficiency of resource allocation and achieve rapid and optimal allocation of resources, so as to alleviate the compensation effect of capital and labour mismatch. Through intelligent collaborative management, enterprises can achieve "double improvement" in production efficiency and environmental protection. Based on the above analysis, this paper proposes the following hypothesis:

H2: Voluntary environmental regulation in agribusiness can improve total factor productivity by driving digital transformation.

### 3.3 Government action, voluntary environmental regulation and total factor productivity

As the two main subjects of environmental regulation, there is a balance between government and business [63]. For the government, it closely links environmental management system certification with the environmental performance of enterprises [8], The government expects companies to be able to achieve certification of their environmental management systems and to have certain requirements, which in turn encourages to raise their awareness of environmental governance, actively take environmental responsibility [64] and reduce environmental pollution. For enterprises, in order to obtain relevant resources and financial support from the government [65] and to build a good corporate image and reputation, they will actively respond to the call of the government’s environmental regulation policy, sending a signal to the community that they have a strong willingness and ability to develop sustainably [66], and actively adopt some voluntary environmental regulatory behaviour.

For agricultural enterprises, its development is not only affected by its own production activities and management mode, but also faces the constraints of government and public supervision [67]. And enterprises should adjust the input of production factors to the optimal state in accordance with the regulations, so as to maintain their production profits, in order to ensure stable growth. In regions where the government pays more attention to environmental protection, based on the signaling theory, in order to obtain strong support from the government and relevant policies, establish a good corporate image and maintain corporate reputation [68], enterprises will actively respond to the government’s call and have more motivation to continuously promote the development of their enterprises in the direction of greening, improving their own environmental management and total factor productivity. In addition, in order to cope with the external green pressure from the government on corporate environmental governance, companies will give more consideration to environmental governance in their strategic planning [69], and as a result, companies will strengthen the development and application of green technologies, green products and green services in their strategic arrangements [70], and urge and motivate companies to implement green innovation, thereby improving environmental performance and total factor productivity. Based on the above analysis, this paper proposes the following hypothesis:

H3: Government environmental concerns play a positive moderating role in the impact of voluntary environmental regulations on total factor productivity of agricultural firms.

**Fig 1:**
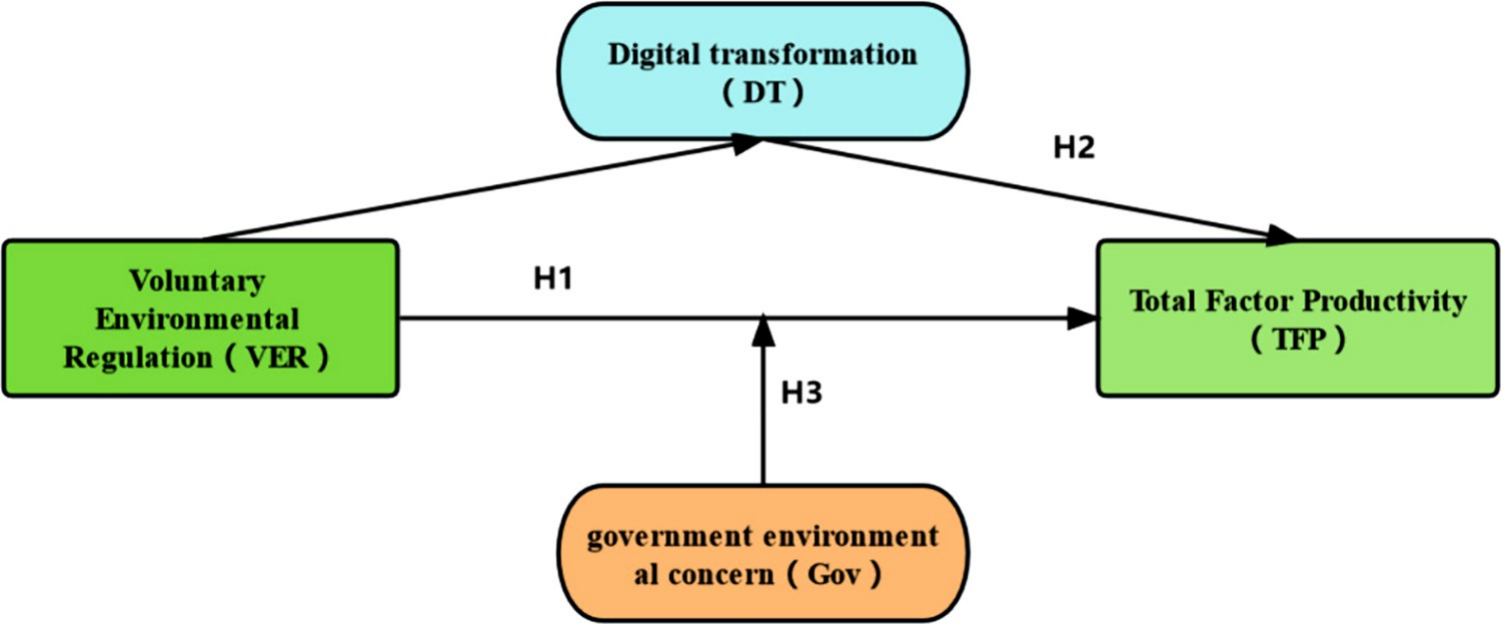
Research theoretical model.

Based on the hypothesis development, we can construct the theoretical model shown in Fig 1.

## 4. Data and variables

### 4.1 Data collection

In this study, China’s listed agricultural companies are selected as observation samples for the following reasons: firstly, from the perspective of globalization, in order to protect human health and social sustainable development. Agricultural environmental issues have no national boundaries, and the quality and safety of agricultural products, soil pollution issues and aggro-ecological environment are generally taken seriously by all sectors of society. Secondly, agriculture is a vulnerable industry with low comparative interests, and agribusinesses engaged in the production and operation of agricultural products are exposed to both natural and market risks. The production activities of agricultural enterprises are extremely dependent on the agricultural environment, and the management of the agricultural ecological environment is of great significance to the sustainable development of agriculture. Thirdly, technological innovation in agribusiness is an important route to productivity improvement, and technological innovation in agriculture is more difficult to develop and apply than technological innovation in other sectors such as industry. The current digital context provides a new historical opportunity for technological change in agribusiness.

Based on the “Guidance on Industry Classification of Listed Companies” revised by China Securities Regulatory Commission in 2012, this paper selects A-stock agricultural listed companies in Shanghai and Shenzhen that focus on agriculture, forestry, animal husbandry and fishery. It also excludes companies with ST, *ST, PT treatment and terminated listing and incomplete information. Finally, 61 listed agricultural companies were selected as the study sample. The data sources mainly include four parts: firstly, data on voluntary environmental regulations are obtained from the National Certification and Accreditation Information Public Service Platform on the official website of the State Administration of Market Supervision and Administration. Secondly, an index reflecting the degree of digital transformation of listed companies, which is obtained by collecting and collating annual reports from 2010–2019 through textual analysis. The third is an index reflecting the extent of the government’s concern for environmental protection, obtained by collecting and collating government work reports from 2010–2019 from the provinces where the enterprises are located, through text analysis method. The fourth is the construction of total factor productivity of listed companies and other micro data at enterprise level from Wind and CSMAR.

### 4.2. Variable measure

#### 4.2.1 TFP

The explanatory variable is TFP, which is a key factor in measuring the extent of the contribution of technological progress to economic growth. At the macro level, domestic and international scholars mainly use methods such as DEA and OLS to measure total factor productivity. At the micro enterprise level, the main methods for measuring total factor productivity include fixed effects method, GMM method, SFA method and semi-parametric estimation method. This paper considers total factor productivity as the difference between the planned and actual output of production, given that the factor inputs used remain constant. When a firm’s factors of production are effectively utilized, its total factor productivity will be higher, resulting in higher economic efficiency. In the empirical analysis, in order to avoid reverse causality and sample selectivity bias, this study mainly selects Levinsohn and Petrin’s [71] method (LP method) to measure total factor productivity of agricultural listed companies. Meanwhile, the OP method [72] was used in the robustness check section to measure the total factor productivity of the firms. Based on the LP method, the production function of the firm is constructed as follows:

$$\ln Y_{\mathrm{it}}=\beta_{0}+\beta_{1}\ln L_{it}+\beta_{2}\ln K_{it}+\beta_{3}\ln M_{it}+\varepsilon_{it}$$ (1)

Where Y is the output of the company, i.e. the ratio of main business income to the value added of the company; L is labour input, i.e. the number of employees of the company; K is capital input, i.e. the net fixed assets of the company; M is intermediate input, i.e. the cash paid by the company to purchase goods and receive services; and εis a random disturbance term.

#### 4.2.2 VER

The core explanatory variable is Voluntary Environmental Regulation (VER), and ISO14001 is used to measure VER. ISO14001, an international standard for environmental management systems developed by the International Organization for Standardization [73], is currently the most comprehensive and systematic international standard for environmental management in the world [74, 75], and is the voluntary environmental regulation program with the largest number of participating companies. Officially certified ISO14001 environmental management systems are valid for three years, with two annual audits and inspections. Specifically, if a company obtains ISO14001 environmental certification in a given year, the value of voluntary participation in environmental regulation for that year and the following two years is 1, otherwise it is 0.

#### 4.2.3 DT

**Fig 2:**
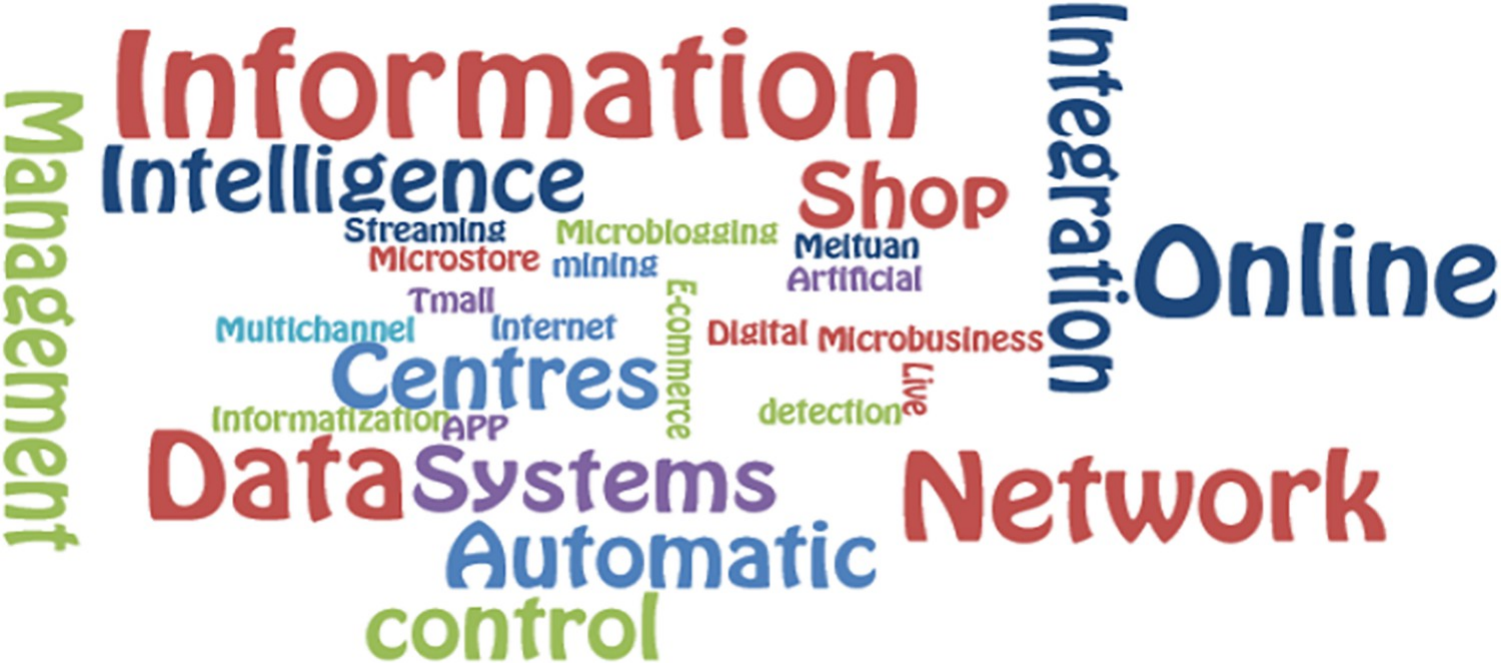
Digital transformation word cloud diagram.

The mediating variable is the Digital Transformation of companies. The annual report of listed companies has the nature of guidance and summary, and it is practical and feasible to use the frequency of key words in annual reports to analyse the level of attention and investment of companies in a certain area. This study uses python crawler technology to collect and collate the text of annual reports of sample companies. Then the annual report text was divided into words through python’s jieba library, and a custom dictionary for this paper was formed based on the results of the division and the research results of scholars (see Table 1 for details). Based on the custom dictionary, the annual report text of each enterprise was analyzed by word frequency statistics, and finally the total word frequency was logarithmically processed to obtain the index of the degree of digital transformation of enterprises. And this paper plots word clouds based on the results of word frequency statistics. The graph shows the frequency of occurrence of keywords related to digital transformation. The results are shown in Fig 2. And in the robustness test, the proportion of items related to digital transformation in the year-end intangible asset details disclosed in the notes of the enterprises’ financial reports to the total intangible assets was used to measure the degree of digital transformation of the enterprises.

**Table 1:** Enterprise digital transformation custom dictionary.

Category	
Digital technology applications	Digitization, big data, cloud computing, cloud IT, cloud ecology, cloud services, cloud platform, block-chain, Internet of Things, data management, data mining, data network, data platform, data centre, data science, digital control, digital technology, digital communication, digital network, digital intelligence, digital terminal, digital marketing, machine learning
Digital Smart Manufacturing	CNC, intelligence, integration, integration, industrial cloud, virtualization, artificial intelligence, high-end intelligence, industrial intelligence, mobile intelligence, intelligent control, intelligent terminal, intelligent mobile, intelligent management, intelligent factory, intelligent logistics, intelligent manufacturing, intelligent storage, intelligent technology, intelligent equipment, intelligent production, automatic control, automatic monitoring, automatic monitoring, automatic inspection, automatic production, integrated control, integrated systems, factory of the future, virtual manufacturing, integrated solutions, lifecycle management, manufacturing execution systems
Digital Information System	Information, networking, information sharing, information management, information integration, information software, information system, information network, information terminal, information centre, industrial information, industrial communication, intelligent networking, intelligent system, mobile internet, industrial internet, industrial internet, internet solutions, internet technology, intelligent fault diagnosis
Digital business models	e-commerce, e-commerce, internet +, online and offline, internet thinking, internet action, internet business, internet mobile, internet application, internet ecology, internet marketing, internet strategy, internet platform, internet model, internet business model, online to offline, online and offline, Internet, O2O, B2B, C2C, B2C, B2B
Digital Operations Channels	multi-channel, omnichannel, new channel, online, onlineization, small program, lead generation, live, anchor, microblog, blogger, circle of friends, mass review, Meituan, hungry, Tmall, Koubei, Xiaohongshu, Today’s headlines, micro shop, micro business, network, online, online shopping, self media, new media, public number, online mall, online offline integration, online offline integration, digital channel mobile, APP, ERP.CRM

#### 4.2.4 Gov

The moderating variable is government environmental concern (Gov). The frequency of occurrence of environment-related terms in provincial government work reports was selected as an indicator of the degree of government environmental concern, referring to Chen and Chen [76], and selected environment-related terms included: environmental protection, pollution, energy consumption, emission reduction, emissions, ecology, green, low carbon, air, chemical oxygen demand, sulphur dioxide, carbon dioxide, PM10, and PM2.5.

#### 4.2.5 Control variables

In order to more accurately analyse the actual impact of voluntary participatory environmental regulation on the total factor productivity of agricultural enterprises, and drawing on existing research in the relevant literature, the following control variables are selected to control for basic enterprise characteristics, operating characteristics and governance characteristics: Size, the logarithm of the firm’s assets per capital assets; Age, the natural logarithm of the number of years of establishment; Roa, the ratio of net profit to total assets; Lev, the ratio of total liabilities to total assets; Intensity, the ratio of total assets to operating income; Sconcention, the proportion of shares held by the largest shareholder; and Board, the logarithm of the number of board members in the company. The variables and their definitions are shown in Table 2.

**Table 2:** Measurements of variables.

Variable type	Variables	Measuring methods
Dependent variable	TFP	Measured according to the LP method
Independent variable	ISO	ISO14001 Environmental System Certification
Mediating variable	lnDig	Analysis based on the text of the annual report
Moderating variable	Gov	Analysis based on the text of the government work report
Control variables	Size	Logarithm of assets per head of business
	Age	Natural logarithm of the number of years of establishment
	Roa	Ratio of net profit to total assets
	Lev	Ratio of total liabilities to total assets
	Intensity	Ratio of total corporate assets to operating income
	Sconcention	Percentage of shareholding of the largest shareholder
	Board	Logarithm of the number of directors of the company

## 5. Empirical results analysis

### 5.1 Principal regression model

In this paper, the empirical testing work has been carried out with the help of stata statistical analysis software. Based on the baseline econometric analysis, we used ordinary least squares(OLS) regression model and random effect model to regress the voluntary environmental regulation and total factor productivity of agribusiness. Among them, the OLS regression model is the basic form of linear regression model, which tries to find the best-fit straight line between the independent variable and the dependent variable. In Stata, the ‘reg’ command can be used to fit the OLS model. Random effects models are suitable for inferring aggregate characteristics from samples. The Hausman test concludes that the random effects model is appropriate for the study in this paper. Meanwhile, in order to solve the problem of omitted variables that vary with individuals and over time, this paper controls for individual random effects and time fixed effects in the empirical evidence. In Stata, the random effects model can be fitted using the random option in the ‘xtreg’ command.

In order to explore the impact of voluntary environmental regulations on total factor productivity of agricultural enterprises, the following benchmark econometric model is developed:

$$TFP_{i,t}=\alpha +\alpha_{1}ISOl_{i,t}+\alpha_{2}X_{i,t}+\mu_{i}+\lambda_{t}+\varepsilon_{i,t}$$ (2)

where TFP denotes the total factor productivity of firm; ISO denotes voluntary environmental regulation of firm; X is a control variable,μis the individual firm effect,λis a time fixed effect andεis a random error term. The parameter α1 indicates the effect of voluntary environmental regulation on the total factor productivity of the firm, and hypothesis 1 holds if α1 is significantly positive.

### 5.2 Principal regression result

Table 3 reports regressions of the impact of voluntary environmental regulations on the total factor productivity of agribusinesses. In this paper, by adding control variables to obtain robust regression results. In the OLS model, column (1) of Table 3 shows that voluntary environmental regulation has a significant positive effect on total factor productivity of agribusiness without adding control variables. Column (2) shows the case where control variables are added and the regression results obtained are consistent with column (1). Column (3) controls for year and individual fixed effects on the basis of column (2), and the regression results still show that voluntary environmental regulation has a positive effect on agribusiness total factor productivity. In the random effects model, columns (4) and (5) of Table 3 show the regression results without and with control variables, respectively, and the regression results show that voluntary environmental regulation has a positive impact on agribusiness total factor productivity. Column (6) controls for year and individual fixed effects and the regression results remain consistent. This suggests that voluntary environmental regulation contributes to the total factor productivity of firms, validating Hypothesis 1.

**Table 3:** Regression results on the impact of voluntary environmental regulation on total factor productivity.

Variable	(1)	(2)	(3)	(4)	(5)	(6)
	OLS			RE		
	TFP	TFP	TFP	TFP	TFP	TFP
ISO	0.592***	0.548***	0.367*	0.371**	0.321*	0.367**
	(0.205)	(0.178)	(0.187)	(0.187)	(0.177)	(0.187)
Control Variable	No	Yes	Yes	No	Yes	Yes
Firm	No	No	Yes	No	No	Yes
Year	No	No	Yes	No	No	Yes
_cons	5.939***	6.413***	9.208***	5.979***	5.722***	9.208***
	(0.090)	(1.429)	(2.424)	(0.212)	(1.627)	(2.424)
N	592.000	583.000	583.000	592.000	583.000	583.000
R^2^	0.014	0.299	0.764	0.005	0.241	0.281

*,**,*** denote significant at 10%, 5% and 1% level respectively, and firm cluster standard error is in parentheses.

All of the above regression results show that voluntary environmental regulation can increase the total factor productivity of agribusiness. According to Porter’s hypothesis theory, in the short term, in order to pass the environmental management system certification, enterprises will increase the cost of fighting pollution and other environmental problems, which directly increases the cost of enterprises. However, in the long term, enterprises will also pursue their own profit maximization while meeting the environmental regulatory standards, which will improve their competitiveness through their own R&D upgrading and technological innovation. Enterprises will also improve their productivity through technological upgrades in the production chain to increase their output and profits. The implementation of voluntary environmental regulation by enterprises will not only strengthen environmental management, but also increase their own productivity, thus realizing a win-win situation for both the environment and the economy.

### 5.3 Mechanism analysis model

After investigating the direct impact of voluntary environmental regulation on total factor productivity of agribusiness, we further explore the path of voluntary environmental regulation on total factor productivity of agribusiness, i.e. the mechanism test analysis in this paper. This paper mainly carries out the mechanism analysis by stepwise test method. First, we test whether voluntary environmental regulation has a significant effect on enterprise digital transformation through regression analysis, and if the regression coefficient is significant, we continue the next test. Adding the variable of enterprise digital transformation in the causal chain, when controlling the variable of voluntary environmental regulation, it still significantly affects enterprise total factor productivity, that is, enterprise digital transformation is the mediating variable in it.

Previous studies have shown that, in terms of enterprise production, digital transformation helps drive technological innovation and intelligent production, enabling the interconnection of data between equipment and systems, thereby improving productivity and resource utilization; in terms of enterprise management, digital transformation helps drive collaborative enterprise management, increasing synergy between levels within the enterprise and reducing information and communication costs, i.e. digital transformation has a positive effect on improving total factor productivity of enterprises. In this study, to examine whether voluntary environmental regulation can increase TFP by driving digital transformation in enterprises, a mediating effect model is constructed on the basis of model (2) as follows:

$$D{\mathrm{igital}}_{i,t}=\beta +\beta_{1}ISO_{i,t}+\beta_{2}X_{i,t}+\mu_{i}+\lambda_{t}+\varepsilon_{i,t}$$ (3)

$$TFP_{i,t}=\alpha +\theta_{1}{\mathrm{ISO}}_{i,t}+\theta_{2}Digital_{i,t}+\theta_{3}X_{i,t}+\mu_{i}+\lambda_{t}+\varepsilon_{i,t}$$ (4)

where Digital denotes the degree of digital transformation of firm; β1 denotes the effect of voluntary environmental regulation on the digital transformation of the firm, θ2 denotes the effect of digital transformation of the firm on total factor productivity, and θ1 denotes the effect of voluntary environmental regulation on total factor productivity after controlling for the digital transformation of the firm. Hypothesis 2 holds when the parameters β1, θ1 and θ2 are significantly positive, indicating that the digital transformation of enterprises can increase the effect of voluntary environmental regulations on total factor productivity.

### 5.4 Mechanism analysis result

The mediating effect of digital transformation of firms in the role of voluntary environmental regulation on total factor productivity in agribusiness was analyzed based on the stepwise test. The results are shown in Table 4. In the OLS model, column (1) of Table 4 reflects a significant positive effect of voluntary environmental regulation on digital transformation of enterprises. Column (2) reflects that the mediating effect exists and the coefficient is significantly positive. The regression results in the random effects model in columns (3) and (4) are also consistent with expectations. Thus, it is shown that digital transformation of firms is a mediating variable in the effect of voluntary environmental regulation on total factor productivity of agribusiness. Hypothesis 2 was tested.

**Table 4:** Mechanisms of digital transformation analysis.

Variable	(1)	(2)	(3)	(4)
	OLS		RE	
	lnDig	TFP	lnDig	TFP
ISO	0.299***	0.505***	0.406***	0.300*
	(0.085)	(0.179)	(0.099)	(0.187)
lnDig		0.165*		0.273***
		(0.087)		(0.087)
Control Variable	Yes	Yes	Yes	Yes
Firm	Yes	Yes	Yes	Yes
Year	Yes	Yes	Yes	Yes
_cons	2.096***	6.154***	2.384***	5.298***
	(0.685)	(1.436)	(0.085)	(0.304)
N	581	581	606	590
r2	0.081	0.304	0.029	0.022

*,**,*** denote significant at 10%, 5% and 1% level respectively, and firm cluster standard error is in parentheses.

The theoretical inference of environmental regulation on total factor productivity under the digital transformation path of firms is further validated empirically through the test of mediating effects. Voluntary environmental regulation by firms can accelerate the process of digital transformation of firms and thus increase TFP. Voluntary environmental regulation behaviour of firms induces them to engage in technological innovation, adopt digital production methods, integrate information technology with agricultural production, and promote the development of digital agriculture and smart agriculture. Thus Hypothesis 3 is tested. Voluntary environmental regulation is an expression of social responsibility and implies a range of technical and managerial improvements in environmental protection. While making it more costly for businesses in the short term, it is beneficial for green and sustainable development in the long term. By promoting the digital transformation of enterprises, it leads to technological progress and TFP improvements.

### 5.5 Moderating effect model

Having analyzed the internal mechanisms at work, we turn to the effect of external factors on the relationship between voluntary environmental regulation and total factor productivity. The moderating effects model is suitable for data analyses that assess whether the effect of the independent variable on the dependent variable depends on other factors. That is, it is assumed that there is a linear relationship between the independent variable and the dependent variable, but the relationship may vary across subgroups (or conditions). In an empirical test, if there is a significant interaction between the independent variable and the moderator variable, it means that the effect of the independent variable on the dependent variable is influenced by the moderator variable.

Therefore, this paper mainly wants to explore what role government behaviour plays in this, and a moderating effects model is used to test whether different government behaviors affect the relationship between voluntary environmental regulation and firms’ total factor productivity. Based on model (2), the interaction term between government action and voluntary environmental regulation is introduced and the model is constructed as follows:

$$TFP_{i,t}=\omega_{0}+\omega_{1}ISO_{i,t}+\omega_{2}Gov_{i,t}+\omega_{3}ISO_{i,t}*Gov_{i,t}+\omega_{4}X_{i,t}+\mu_{i}+\lambda_{t}+\varepsilon_{i,t}$$ (5)

where Gov denotes the degree of government environmental concern in firm’s province. ISO*Gov represents the implication that hypothesis 3 holds when the interaction term parameter ω3 is significantly positive, indicating that government environmental concern enhances the contribution of voluntary environmental regulation to total factor productivity.

### 5.6 Moderating effect result

The effect of the government’s level of environmental concern on the effect of voluntary environmental regulation on the role of total factor productivity in agribusiness was analyzed according to the moderating effects model. The results are shown in Table 5. In the OLS model, column (1) of Table 5 shows the regression results with control variables added, in which the interaction term between voluntary environmental regulation and the degree of government’s environmental concern is significantly positive, reflecting that the government’s behaviour plays a positive moderating role between voluntary environmental regulation and agribusiness total factor productivity. Column (2) controls for individual and year fixed effects of firms on the basis of column (1), and the results are consistent with column (1). In the random effects model, the interaction term between voluntary environmental regulation and the level of government environmental concern is still found to be significantly positive through columns (3) and (4). Thus, Hypothesis 3 of this paper is tested.

**Table 5:** Analysis of the moderating effects of government action.

Variable	(1)	(2)	(3)	(4)
	OLS		RE	
	TFP	TFP	TFP	TFP
ISO	-1.089**	-0.765	-1.089**	-0.765
	(0.550)	(0.567)	(0.550)	(0.567)
gov	34.281	48.854	34.281	48.854
	(35.836)	(40.164)	(35.836)	(40.164)
ISO_gov	203.844***	166.526**	203.844***	166.526**
	(76.531)	(77.628)	(76.531)	(77.628)
Control Variable	Yes	Yes	Yes	Yes
Firm	No	Yes	No	Yes
Year	No	Yes	No	Yes
_cons	5.691***	9.481***	5.691***	9.481***
	(1.617)	(2.417)	(1.617)	(2.417)
N	583	583	583	583
r2	0.257	0.2965	0.257	0.296

*,**,*** denote significant at 10%, 5% and 1% level respectively, and firm cluster standard error is in parentheses.

In the process of the impact of corporate environmental governance on its total factor productivity, according to signaling theory, the government’s degree of concern for environmental protection enhances the intensity of corporate voluntary environmental regulations as a way to increase corporate total factor productivity. This argument is also validated by the results of the analysis of the moderating effects of government actions according to Table 5. It suggests that the operation of voluntary environmental regulations on firms’ total factor productivity is stronger in provinces with higher government environmental concerns compared to provinces with lower government environmental concerns, i.e. firms’ awareness of environmental governance is strengthened under the government’s external regime, and government environmental concern will promote the effect of voluntary environmental regulation on total factor productivity of firms, verifying hypothesis 3.

### 5.7 Robust analysis

To ensure the reliability of the findings, the following robustness tests were also conducted in this paper. The first is the replacement of the explanatory variables. The OP method (Olley and Pakes, 1996) is used to measure the total factor productivity of firms. Olley—Pakes uses a semi-parametric estimation method in which the amount of investment in the firm is introduced into the equation as a proxy variable for the firm’s observable efficiency, which can better deal with the endogeneity problem caused by sample selection bias. The estimation results are shown in Columns (1) and (2) of Table 6. The results show that the positive effect of voluntary environmental regulation by firms on total factor productivity remains significant after replacing the measurement of the explanatory variables, consistent with the main regression results, and hypothesis 1 is still supported without changing the basic conclusions.

**Table 6:** Robustness test analysis.

Variable	(1)	(2)	(3)	(4)	(5)	(6)
	OLS	RE	OLS		RE	
	TFP_OP	TFP_OP	DT	TFP	DT	TFP
ISO	0.374*	0.374*	0.034**	0.490***	0.054***	0.320*
	(0.196)	(0.196)	(0.016)	(0.178)	(0.016)	(0.189)
DT				1.335***		0.907*
				(0.465)		(0.498)
Control Variable	Yes	Yes	Yes	Yes	Yes	Yes
Firm	Yes	Yes	Yes	Yes	Yes	Yes
Year	Yes	Yes	Yes	Yes	Yes	Yes
_cons	9.095***	9.095***	-0.461***	6.997***	0.088***	5.908***
	(2.548)	(2.548)	(0.129)	(1.441)	(0.018)	(0.217)
N	583	583	577	577	586	586
r2	0.267	0.267	0.097	0.311	0.021	0.011

*,**,*** denote significant at 10%, 5% and 1% level respectively, and firm cluster standard error is in parentheses.

The second is the replacement of mediating variables. In fact, in existing studies, text analysis has been widely used to measure the level of digital transformation of enterprises, this method, to a certain extent, portrays the level of digitization of the company, but relying only on text mining to extract and analyse keywords lacks professional judgment, and it cannot reflect the practical process of digital transformation of enterprises comprehensively and accurately. In this paper, we use the proportion of items related to digital transformation in the year-end intangible assets breakdown disclosed in the notes of enterprises’ financial reports to measure the degree of digital transformation of enterprises, and the estimation results are shown in columns (3) to (6) of Table 6, which shows that the mediating role of enterprises’ digital transformation is still significant. The results show that the mechanistic role of corporate digital transformation in this remains significantly positive after replacing the measurement of mediating variables, consistent with the previous regression results.

## 6. Conclusions and further discussion

High-quality development must be green and sustainable development. Accelerating the construction of ecological greening and forming green production methods will lead to the continuous improvement of environmental quality, which can also provide more space for economic development. Based on a theoretical analysis of the relationship between voluntary environmental regulation and total factor productivity of agricultural enterprises, this paper conducted an empirical test on the panel data of 61 Shanghai and Shenzhen A-share listed agricultural enterprises from 2010 to 2019, and finally obtained the following conclusions: firstly, through the results of benchmark regression, voluntary environmental regulation has a significant promotion effect. This provides empirical evidence that firms achieve both environmental governance and productivity gains. Secondly, the mediating effects model was used to verify the role of the digital transformation process in voluntary environmental regulation and total factor productivity. The results show that voluntary environmental regulations force firms to accelerate their digital transformation process and improve their own total factor productivity through digital management and technological progress. This, to a certain extent, validates and enriches the innovation compensation effect of Porter’s hypothesis. Finally, the impact of government action in this is empirically analyzed through a model of the moderating effect of government environmental concerns. The degree of government concern for environmental protection promotes the positive effect of voluntary environmental regulation on total factor productivity of agricultural enterprises. The emphasis on environmental aspects in the government’s work report increases the willingness of firms to manage their own environment, which in turn improves their environmental performance and total factor productivity. Thus, on the whole, voluntary environmental regulation has a significant positive effect on the innovation compensation effect of agribusinesses, and these findings are important for the sustainable development of agribusinesses.

Based on the results of the study, this paper puts forward the following policy recommendations. In terms of enterprises, firstly, voluntary environmental regulation is more flexible and autonomous than command-and-control or market-incentive environmental regulation, and it is important for enterprises to promote green development on their own. From the perspective of sustainable development, as environmental management will increase the cost of management, enterprises should combine their environmental management with their innovation and digital management capabilities to reduce costs and increase efficiency, so as to improve their total factor productivity and achieve sustainable development. Secondly, in the current context of digitization, agricultural enterprises can integrate digital technologies such as big data and cloud computing into the agricultural production process, with the help of intelligent algorithms and data to guide enterprises to improve production plans, enhance the operational efficiency of equipment, optimized production plan, avoid waste of resources and pollutant emissions, and at the same time improve resource utilization, maximize the technological advantages brought by digital transformation to enterprises, and achieve technological upgrading and this will maximize the technological advantages of digital transformation and achieve a win-win situation for both technological upgrading and environmental management.

On the government side, firstly, agricultural development relies on the agricultural environment. In view of the current situation of agricultural surface pollution, the government should increase the monitoring of agricultural land protection, establish a sound land science and technology innovation system that combines with arable land protection, green development and ecological restoration to ensure sustainable land use and provide a good prerequisite for agricultural development. Secondly, the government should provide good policy environment support for agricultural enterprises to promote digital and green development. In terms of policy, appropriate documents and policies should be issued to improve the system and management of voluntary environmental regulations for enterprises. In terms of government subsidies, the government should provide subsidies and incentives for the digitization process and green development of enterprises, so as to promote enterprises to shift from traditional innovation to green innovation and upgrade, and at the same time, take into account the differences in total factor productivity and digitization process of agricultural enterprises, so that enterprises with lower productivity can accelerate their digital transformation process. Thirdly, the government should play a leading role in the prevention and control of agricultural pollution and the sustainable development of the land. On the one hand, we should rely on the relevant government departments to strengthen publicity and guidance, strengthen the public’s awareness of green production and pollution prevention through special lectures and seminars, promote the formation of a sustainable soil management mechanism and implement the concept of sustainable land development. The concept of sustainable use should be incorporated into regular management, thus improving the quality and efficiency of pollution management in general.

This study analyses the effect of voluntary environmental regulation and total factor productivity of agricultural firms at the micro level, and provides empirical suggestions for managers to optimize their decisions and improve productivity. However, voluntary environmental regulations are the result of firms’ self-selection, and there may be selection bias in the empirical evidence. In the follow-up study, we hope to solve the endogeneity problem in the study as far as possible through econometric methods, in order to improve the relevant research and make better suggestions for firms and governments.

## Supporting information

S1 Data (XLSX)
